# Ambulance clinicians implementing evidence-based practice: mind the gap! Attitudes, perceptions and experiences of student paramedics

**DOI:** 10.29045/14784726.2023.12.8.3.11

**Published:** 2023-12-01

**Authors:** Jon Newton, Andrew D. A. C. Smith

**Affiliations:** University of the West of England ORCID iD: https://orcid.org/0000-0001-5407-0694; University of the West of England ORCID iD: https://orcid.org/0000-0001-5452-9901

**Keywords:** education, evidence-based practice, implementation science, paramedic

## Abstract

**Background:**

Evidence-based practice (EBP) represents the conscientious and judicious use of the best contemporaneous evidence in partnership with patient values and clinical expertise to guide healthcare professionals. As a result, EBP is a recommended component of undergraduate education and considered fundamental for improving patient outcomes. EBP principles have thus become deeply rooted in higher education curricula, but only in recent years has this begun to permeate the world of paramedic practice. Despite this paradigm, the impact of EBP may be limited because ambulance clinicians may struggle with implementation, as a variety of barriers influence translation and application.

**Methods:**

A survey study aimed to gain insight into the epistemological and metacognitive barriers impacting student experience in order to help improve teaching and learning practices.

**Results:**

A sample of 64 students, across two different undergraduate paramedic science programmes, were recruited. Of these, 70% of BSc (Hons) students versus 33% of DipHE students agreed to some extent or greater that EBP represented minimal benefit in real-world practice due to Trust policy and the guidelines set out by the Joint Royal Colleges Ambulance Liaison Committee (Welch’s t = 2.571, df = 26, p = 0.016 two-sided). Furthermore, 25% felt standard operating procedures negatively impacted their ability to implement EBP, and 39% reported their EBP learning had improved their ability to implement improved levels of patient care.

**Conclusion:**

A disparity between theoretical learning and EBP implementation was identified. EBP may not dovetail with standard operating procedure within UK ambulance Trusts, resulting in confusion among student paramedics as to the true worth of EBP.

## Introduction

Evidence-based practice (EBP) is regarded as the conscientious and judicious use of the best contemporaneous evidence in partnership with patient values and clinical expertise to guide healthcare professionals (Titler, 2008); yet, prior to the introduction of degree-level paramedic education, EBP was not widely taught to ambulance service trainees (Emms & Armitage, 2010). Over the last decade, universal incorporation of EBP into UK curricula has propelled clinical and scholarly development, and the shift from protocol-led treatments to independent decision-making is now underpinned within higher education (European University Association, 2022). EBP is recognised as a cornerstone of clinical effectiveness (Lehane et al., 2020), a recommended component of an undergraduate healthcare education (Lehane et al., 2018) and fundamental for improving healthcare quality as well as patient outcomes (Leufer & Cleary-Holdforth, 2009). Nevertheless, this change in paradigm is still in its infancy, and implementation of EBP may be considered contentious – stemming from institutionalised culture, medico-legal challenges and financial/political conflicts within the ambulance service and the wider healthcare system. Despite these difficulties, educating the future workforce to be autonomous clinicians while meeting the requirements of a growing, changing and ageing population should be considered a foundation of contemporary healthcare education.

Paramedics perceive themselves as generalist clinicians capable of responding to patients of all ages, with any presenting complaint (Eaton et al., 2021), and this is underpinned by the university education students now receive. Yet anecdotal observations suggest EBP implementation by ambulance clinicians is an alien concept to the other healthcare disciplines, perhaps because paramedics can be perceived as little more than ambulance drivers. While it is acknowledged by both the general public and the healthcare system that the paramedic role has evolved significantly since its inception in the 1970s, and that paramedics have continued to become increasingly respected and medicalised healthcare professionals (Brooks et al., 2015), general practitioners (GPs) and accident and emergency staff may not possess a true understanding of the modern paramedic’s skillset.

A study evaluating paramedic practice in primary care settings found ‘role boundaries’ were considered blurred, and push-back from other healthcare professionals related to a lack of trust, feeling threatened or a sense of disempowerment (Eaton et al., 2021). In this setting, some GPs perceived paramedics as being able to offer an ‘eyes and ears’-only approach and did not regard them as autonomous clinicians able to diagnose and manage patients on their own. In the first author’s experience in emergency and critical care settings, the paramedic is rarely perceived as a diagnostician or decision-maker, rather as simply a skilled intermediary forming part of a patient’s journey to definitive care. These viewpoints are representative of a contentious debate and a pertinent cultural challenge.

Paramedics are increasingly tasked with complex decision-making relating to whether to convey a patient to hospital or manage their condition at the scene (Voss et al., 2020). These heterogeneous encounters frequently present within challenging, time-pressured situations (O’Hara et al., 2014), and clinicians are required to tackle moral and ethical dilemmas (Bruun et al., 2022), utilise intuition and draw upon clinical knowledge beyond the guidelines outlined by the Joint Royal Colleges Ambulance Liaison Committee (JRCALC). Utilising EBP at junctures of this nature has become important and is perhaps why research-informed decision-making is now a component in the standards-of-proficiency framework (Health and Care Professions Council, 2023).

Few studies have specifically evaluated the attitudes, perceptions and experiences of student paramedics in clinical practice (or graduates), nor the challenges or successes faced when learning to implement EBP. A systematic review by Saunders et al. (2019) emphasised that while practising healthcare professionals valued EBP for improving care quality, this did not translate into EBP behaviours, and across multiple disciplines implementation in daily practice was generally at a low level (Saunders & Vehviläinen-Julkunen, 2014; Scurlock-Evans et al., 2014; Ubbink et al., 2013; Upton et al., 2014). This multidisciplinary sample also rated their EBP knowledge and skillset at an insufficient level to integrate best evidence into daily practice. This is consistent with the findings of the studies previously conducted by Wallen et al. (2010), Melnyk et al., 2012 and Melnyk et al., 2017, which showed the majority of clinicians do not consistently engage with EBP. Human factors and shortfalls in professional identity may further contribute to this sub-optimal landscape and an unwanted growth in the EBP theory–practice gap.

Developing the future workforce to become autonomous practitioners is perhaps a contemporaneous ideal, because in real-world practice ambulance clinicians face a great many challenges associated with EBP (Emms & Armitage, 2010) from both an educational and operational standpoint – even after they have qualified (Simpson et al., 2012; Warren et al., 2016; Wilson et al., 2021). While higher education establishments continue to seek to provide students with the toolkit for independent learning and opportunities for scholarly advancement (Advance HE, 2022), this is met with duplicity in the workplace and tandem steps to scale back autonomy in exchange for strict, ‘black and white’ algorithmic approaches. The reasons for this are complex, and revolve around risk management, politics and Trust liability, but when it comes to EBP it would appear higher education and ambulance Trusts are pulling in opposing directions.

In the first author’s professional practice, it is not uncommon to witness qualified paramedics supress EBP knowledge and skills in favour of local policy algorithms to guide decision-making. These behaviours are typically due to fear of reprimand for practising outside Trust guidelines and the risks associated with making incorrect decisions. This contributes to negative ambulance service culture, which cascades down to student clinicians and leads to push-back within the higher-education classroom. The result of this disparity between ‘best practice’ and actual clinical care ultimately results in the lowering of clinical standards and slows research and development. A further concerning observation is that undergraduate students tend to favour highly scripted and tangible algorithms over broader learning opportunities as a means of omitting the ‘grey’ areas inherent in modern medicine. Educators can worsen this problem by providing learning materials created simply from local policy algorithms or the JRCALC guidelines. While these resources are typically safe, simple and risk-free, we suggest this approach can limit the cultivation of critical thinking and problem-solving skills, and hinder autonomous, heuristic learning. Students are frequently puzzled by what EBP really represents, and for it to be effectively incorporated they must see this learning as more than an advancement in pure scholarship, and instead recognise its clear link with professional practice (Wilson et al., 2021).

This study aimed to investigate the attitudes, perceptions and experiences of EBP learning and implementation among student paramedics. Differences in patient-facing experience, age and the route taken into higher education exist among student paramedics, and we additionally aimed to identify whether the experience of EBP differed between the students’ programme of study (BSc (Hons) vs DipHE).

## Methods

A survey study was conducted at University of the West of England (UWE) in the 2020–2021 academic year. The potential population were all final-year students on paramedic science programmes. Participants met inclusion criteria if they were a final-year UWE paramedic student on the BSc (Hons) or DipHE programme and were currently completing their paramedic practice portfolio within South Western Ambulance Service NHS Foundation Trust.

Participants were excluded if they had not completed their first-year paramedic practice portfolio. Completion of the portfolio was deemed important because students are required to have clinical skills signed off by practice placement mentors, as well as undertake basic reflective writing and critical reading to enhance knowledge spheres. These criteria ensured all eligible participants had experienced at least 18 months of front-line ambulance service practice. Participants were also excluded if they had not completed the EBP module or had completed it online during the COVID-19 pandemic rather than face to face. We consider this an important exclusion because those learning online had considerably less patient-facing time to implement EBP under supervision and their theoretical learning could not be considered homogenous with those taught face to face. Finally, students who had been personally tutored or mentored in clinical practice by the first author were also excluded. This control measure was added to ensure participants were not subjected to indirect pressures or perceived biases which may have led them to predict or appease the research team within their study questionnaires, and facilitated an increased level of rigour by mitigating for the potential of systematic biases.

**Figure 1:**
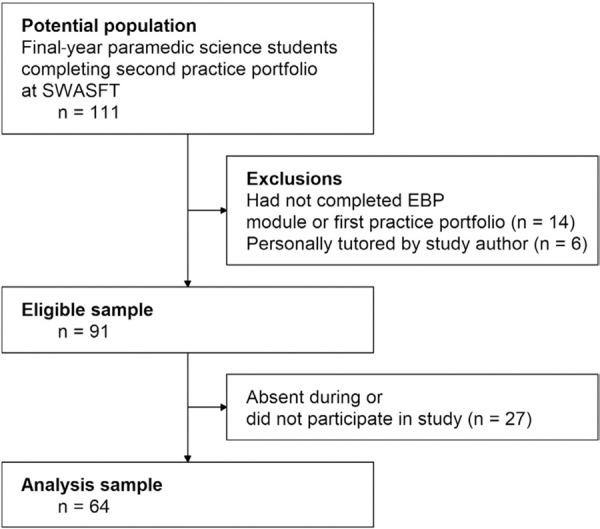
Figure 1. Formation of the analysis sample.

Not all eligible participants could be included in this study due to having to self-isolate, or testing positive, for COVID-19. Some students were also absent from university at the time this study was conducted due to personal circumstances, further reducing the number of eligible participants. The formation of the analysis sample is shown in Figure 1.

Participants in the analysis sample were given a questionnaire to complete, comprising seven statements, and their agreement was recorded using a 7-point Likert scale. For presentation of results, we grouped the 7-point scale into three categories: agree, disagree and undecided. Participation was optional and anonymous, and no financial incentives/rewards for survey completion were available. Those completing the survey did so as an act of goodwill. Data were collected at the same point in time and completed questionnaires were posted into a sealed collection box, enabling views to be expressed without the risk of feeling judged or criticised. Baseline characteristics were also collected to add data richness and minimise assumptions and generalisations. All participants in this study were accounted for and the data were checked prior to analysis by an experienced statistician to ensure responses, figures and numerical values tallied correctly, to improve internal validity and rigour. The acquired data were analysed using IBM SPSS v26 software, and Welch’s t-test (Derrick & White, 2017) was utilised to compare the responses provided by students on the two programmes (BSc vs DipHE). The ‘undecided’ option lay in the middle of the 7-point scale, so it could represent a genuine position of neither agree nor disagree, or it could represent a non-committal answer. This could lead to convenience bias if participants routinely opt to provide a non-committal answer. To ascertain the impact this bias could have, we conducted a sensitivity analysis, repeating our analysis with the undecided responses removed.

## Results

The sample available for analysis consisted of 64 students, with 46 from the BSc (Hons) programme and 18 from the DipHE programme. The characteristics of students in the two programmes are shown in Table 1. Participants from the DipHE programme were more likely to be male, more likely to be aged over 30 years and had prior ambulance service experience.

**Table 1.** Characteristics of the analysis sample of 64 final-year paramedic students.

	Whole sample n = 64	BSc (Hons) paramedic science n = 46 (72%)	DipHE paramedic science n = 18 (28%)
**Sex:**			
Female Male Other options*	39 (61%) 24 (38%) 1 (1%)	32 (70%) 13 (30%) 0 (0%)	7 (39%) 10 (56%) 1 (6%)
**Age:**			
19–30 years 31–40 years 41+ years	45 (70%) 13 (20%) 6 (9%)	43 (94%) 2 (4%) 1 (2%)	2 (11%) 11 (61%) 5 (29%)
**Ambulance experience prior to starting programme:**			
None 1–10 years 2–5 years 6–10 years 11+ years	43 (67%) 2 (3%) 13 (20%) 4 (6%) 2 (3%)	2 (91%) 1 (2%) 3 (7%) 0 (0%) 0 (0%)	1 (6%) 1 (6%) 10 (56%) 4 (22%) 2 (11%)

*‘Other options’ included: transgender; do not define as either; prefer not to say. These categories were grouped before analysis to reduce the likelihood of identifying participants.

Table 2 summarises the responses to the seven statements on the questionnaire. Less than half (39%) of students agreed that the knowledge they had gained from the EBP teaching had improved their ability to implement evidence-based care. A majority (73%) agreed that following their EBP studies they were able to critically appraise medical literature and make safer decisions. Less than half (39%) of students agreed that the EBP module had improved their confidence in preparing them for their role of newly qualified paramedic (NQP). Less than a third (33%) of students agreed they had observed EBP being implemented by their mentors. Less than a fifth (17%) of students agreed that EBP was discouraged by senior managers. A quarter (25%) of students agreed the Trust’s clinical guidelines and standard operating procedures prevented the implementation of EBP. There was a difference between BSc students and DipHE students as to whether EBP represented minimal benefit to paramedics in real-world practice due to the presence of Trust policy and JRCALC guidelines: 70% of BSc students, compared with 33% of DipHE students agreed EBP represented minimal benefit. Removing the undecided results had no impact on results (Supplementary 1).

**Table 2.** Questionnaire responses from 64 final-year paramedic students (46 BSc (Hons) paramedic science students and 18 DipHE paramedic science students).

Statement		Disagree	Undecided	Agree	Average difference BSc – DipHE*
The knowledge you have gained from your EBP module has improved your ability to implement an enhanced level of patient care.	Whole sample	19 (30%)	20 (31%)	25 (39%)	0.075 (t = 0.199, p = 0.843)
	BSc	13 (28%)	15 (33%)	18 (39%)	
	DipHE	6 (33%)	5 (28%)	7 (39%)	
The EBP knowledge you have gained now allows you to critically appraise medical literature, and make safer clinical decisions.	Whole sample	10 (16%)	7 (11%)	47 (73%)	0.418 (t = 1.196, p = 0.241)
	BSc	6 (13%)	4 (9%)	36 (78%)	
	DipHE	4 (22%)	3 (17%)	11 (61%)	
Completing a module in EBP has improved your confidence in helping prepare you for the role of an NQP.	Whole sample	23 (36%)	16 (25%)	25 (39%)	0.176 (t = 0.428, p = 0.672)
	BSc	16 (35%)	11 (24%)	19 (41%)	
	DipHE	7 (39%)	5 (28%)	6 (33%)	
You regularly observe your mentor(s) (and other senior paramedic colleagues) implement EBP in their day-to-day work.	Whole sample	31 (48%)	12 (19%)	21 (33%)	0.548 (t = 1.259, p = 0.217)
	BSc	20 (43%)	9 (20%)	17 (37%)	
	DipHE	11 (61%)	3 (17%)	4 (22%)	
The implementation of EBP is discouraged by ambulance Trust managers.	Whole sample	32 (50%)	21 (33%)	11 (17%)	0.609 (t = 1.515, p = 0.141)
	BSc	21 (46%)	16 (35%)	9 (20%)	
	DipHE	11 (61%)	5 (28%)	2 (11%)	
The Trust’s clinical guidelines and standard operating procedures negatively impact your ability to implement EBP.	Whole sample	28 (44%)	20 (31%)	16 (25%)	0.029 (t = 0.079, p = 0.937)
	BSc	20 (43%)	14 (30%)	12 (26%)	
	DipHE	8 (44%)	6 (33%)	4 (22%)	
EBP offers minimal benefit because Trust policy and JRCALC guidelines provide everything required to support clinical decision-making.	Whole sample	17 (27%)	9 (14%)	38 (59%)	1.302 (t = 2.571, p = 0.016)
	BSc	8 (17%)	6 (13%)	32 (70%)	
	DipHE	9 (50%)	3 (17%)	6 (33%)	

*Average Likert scale response in BSc students minus average Likert scale response in DipHE students, analysed using Welch’s test.

Participants rated their agreement with the statements on a 7-point Likert scale, with responses labelled as 1 = strongly disagree; 2 = disagree; 3 = disagree to some extent; 4 = undecided; 5 = agree to some extent; 6 = agree; 7 = strongly agree. For this table, responses 1, 2, 3 are grouped into ‘disagree’, and responses 4, 5, 6 are grouped into ‘agree’.

EBP: Evidence-based practice; JRCALC: Joint Royal Colleges Ambulance Liaison Committee; NQP: newly qualified paramedic.

## Discussion

We observed that only 39% of the sample felt that the EBP knowledge gained from their university education had resulted in an improved ability to implement evidence-based patient care. The reasons for this are likely multi-factorial, but parallel studies have highlighted that if learners do not embrace the attitudinal and behavioural changes needed within the EBP domain, the acquisition of theoretical skills and knowledge is of little consequence (Ilic & Forbes, 2010; Maggio et al., 2013; Wilson et al., 2015). For some, this will be challenging because of ongoing conflicts within the paramedic profession relating to its status as a professional discipline (Givati et al., 2017). Attitudes to EBP may also be tainted if negative behaviours are demonstrated by mentors, managers or fellow learners, and a student’s view of the paramedic profession will be influenced by the fact the practical skills learnt on undergraduate programmes are those which most clearly resemble clinical practice (Wilson et al., 2015).

It is reasonable to hypothesise that these findings are the consequence of learners failing to acquire a satisfactory grasp of the core EBP learning objectives. However, the wider literature demonstrates that undergraduate students studying an allied health discipline (or medicine) will readily gain skills in literature-searching and develop some analytical abilities (Bozzolan et al., 2014; Coomarasamy & Khan, 2004; Dizon et al., 2012; Ilic & Forbes, 2010; Ilic & Maloney, 2014; McEvoy et al., 2018; Thomas et al., 2010), but struggle with application and implementation. Our findings correspond with this literature, as 73% of our sample felt their EBP studies enabled them to critically appraise medical literature and make safer decisions. However, the notion that EBP may lack tangibility and is thus challenging to extrapolate in a patient-facing capacity is perhaps represented by the fact that only 39% of our sample claimed that their EBP training had improved clinical confidence levels in preparation for an NQP role. It is plausible to suggest these individuals may not have received adequate mentorship, or opportunities to regularly employ EBP during ambulance placement, although this was not a theme specifically explored within this study. The continuing increase in ambulance service demand and pressures to reduce on-scene times (Claridge & Parry, 2021) may also have influenced this result. Improvements as to how mentors construct and implement treatment plans may need to be more robustly explained to learners, with a view to reducing these percentages and improving future practice.

Less than a third of our sample agreed they had observed EBP being implemented by their mentors. This could indicate opposing clinical viewpoints among paramedic mentors within South Western Ambulance Service NHS Foundation Trust as to the benefits of EBP. However, less than a fifth of respondents reported EBP being discouraged by senior managers, which was reassuring and indicative that the majority of students do not actively feel autonomy is being ringfenced at a local level. A quarter of our sample felt ambulance Trust guidelines and standard operating procedures negatively impacted their ability to implement EBP. It is concerning that some students felt clinically compromised in practice, especially at such an early stage of their career. These figures indicate that not all students are routinely utilising EBP to the best of their ability, and this finding correlates with that of the systematic review by Saunders et al. (2019).

We also identified a belief that EBP represented minimal benefit to paramedics in real-world practice due to the presence of Trust policy and JRCALC guidelines. This was more prevalent in BSc (Hons) students than DipHE students. This is the most concerning finding from both an educational and clinical standpoint, because it indicates disparity between (a) the university’s intended learning objectives; (b) paramedic student experience; and (c) the clinical trajectory of the ambulance Trust. The fact that the students on the DipHE programme were comparatively older and significantly more clinically experienced than those on the BSc (Hons) course might explain why a higher number of DipHE students felt EBP represented greater benefits. No significant differences were observed between the two programmes for any other response on the questionnaire. At present there is limited paramedic-specific research on how experience affects EBP implementation, but evidence from the nursing profession indicates that less-experienced staff face more barriers to EBP implementation than their senior colleagues (Ammouri et al., 2014; Oh, 2008; Zhou et al., 2015). A self-perceived lack of authority and power to change care procedures has been identified as the most significant factor preventing nurses from using research results in practice (Pitsillidou et al., 2021). There is also evidence that nurses’ opinions regarding patient care delivery are felt to have less value (Brown et al., 2009), and their autonomy is limited by the higher social status and prerogative knowledge held by doctors (McKay & Narasimhan, 2012). Our results suggest at least some junior ambulance staff reside in a similar position.

The right organisational settings facilitate the translation of evidence into healthcare practice (NHS England & NHS Improvement, 2020), but infrastructure deficiencies remain a significant obstacle across multiple healthcare disciplines (Uysal et al., 2010; Wang et al., 2013). EBP needs to be delivered progressively through a learner’s educational journey and showcased regularly within practice placements to grow confidence in its application (Pitsillidou et al., 2021). When organisations fail to provide these environments, the potential for EBP influence and impact is negated, and its perceived value to operational staff diminished. Our findings correlate with the wider literature and may help explain why so many of the students within our sample felt EBP offered limited benefit in clinical practice. Influencing interprofessional behaviours to cultivate positive change is perhaps optimistic due to the hierarchical structure embedded in the healthcare system, and the timeframes required for EBP integration and implementation (Schaefer & Welton, 2018). Such promotion requires behavioural change by individual clinicians (and their mentors), organisational readiness and a strong partnership with education systems that are sufficiently equipped to deliver EBP competency (Lehane et al., 2018). Those simply paying lip service to EBP risk delivering sub-optimal patient care, increased spending (Pitsillidou et al., 2021) and hindering positive cultural entrenchment at an operational level.

### Limitations

We recognise studies with small sample sizes naturally return data insufficiencies, preventing researchers from obtaining a true estimate of effect (Deziel, 2018). This was mitigated to some extent by sampling from two paramedic programmes. However, reduced student attendance owing to COVID-19 reduced our sample size and this had scope to impact our results. Our analysis sample size gave approximately 70% statistical power to detect a difference between programmes of 1 point on the Likert scale. Therefore, although we were able to detect differences between programmes, which are likely the result of different amounts of prior experience, there may be differences between programmes that we were not able to detect. However, this does not affect our findings regarding the reported experiences of students on both programmes. We do not believe that the missing values resulting from our reduced sample size have significantly biased our results: missingness is due to short-term absence owing to COVID-19 and this is unlikely to be related to EBP perceptions. Data are therefore missing completely at random, and thus bias is not expected to be present (Hughes et al., 2019).

Participants were asked closed questions which corresponded with a numerical score, and because no face-to-face questioning was conducted it is difficult to be certain whether the viewpoints captured in our questionnaire accurately reflect intended opinions. Participants could also have misunderstood the study questions or provided unintentional answers, skewing the analysed data. Moreover, what constitutes EBP is subjective, and participants may have possessed differing views. Our study design featured no free-text boxes in the questionnaire nor any other instruments, thereby preventing a supporting qualitative investigation. It is also important to appreciate that these results are limited to this study population and may not represent the attitudes, perceptions and experiences of the remaining population of paramedic students, or those working in other ambulance Trusts.

While all questionnaires were completed simultaneously to reduce the likelihood of bias, it is possible documented responses could have been peer-guided, perceived cultural beliefs of the Trust, or by rare/standout incidents or events. A large proportion (23%) of responses by study participants were ‘undecided’. Participants may have chosen this as a non-committal answer or ‘easy’ option, or to oppose any perceived angle they felt the researchers may have. This could lead to convenience bias. However, our sensitivity analysis removing the undecided responses made no change to our results, hence we do not believe the undecided option has sufficient leverage in our data to cause bias.

Finally, the collected data were interpreted without a screening process to review the questionnaires and ensure each student had answered as intended (i.e. prior to the data being sent for analysis). This had the potential to impact reliability and internal validity. Despite these limitations, high levels of rigour were maintained throughout to ensure the study was objectively executed, fair on all levels and each participant was treated equally.

## Conclusion

A significant proportion of paramedic students in this study felt EBP offered minimal benefit to their clinical practice. Further research is required to ascertain if this viewpoint is representative of the wider student paramedic population within UK ambulance services. A disparity between theoretical learning and its application in practice was also apparent. It would appear that EBP does not seamlessly dovetail with standard operating procedures, and this may be responsible for causing confusion among student paramedics as to the subject’s true worth.

Given the small-scale nature of this study and its associated limitations, no fundamental changes to the way EBP is implemented in practice-based learning settings are justifiable at this time. However, from an educational standpoint these findings should be considered worrisome, and paramedic lecturers, Trust mentors and policy makers may wish to pause for reflection. Deconstructing how EBP is taught, applied and integrated into practice is however warranted. Further research into this aspect of curriculum delivery and extrapolation in a real-world context will hopefully lead to wider professional understanding, improved student experience and innovations in teaching and learning practices.

## Acknowledgements

We would like to thank the student clinicians at University of the West of England who took part in this study.

## Authors contributions

JN designed, wrote and produced the manuscript, and ADACS conducted the data analysis. JN acts as the guarantor for this article.

## Availability of data and materials

The data that support the findings of this study are available from the corresponding author upon reasonable request. All data associated with this manuscript have been stored in a repository at University of the West of England.

## Conflict of interest

None declared.

## Ethics

All methods were carried out in accordance with the Declaration of Helsinki, as well as all other relevant guidelines and regulations. Ethical approval and consent to participate was reviewed by the Institutional Research Committee at University of the West of England and approved as an exempt study, with an informed consent waiver provided due to the observational nature of the study and the involvement of normal educational practices. Therefore, participants were not consented because they were participating in routine debriefing practices as part of standard curricula.

## Funding

None.
